# Psychiatric Disorder and Incessant Tachyarrhythmia in a Child

**DOI:** 10.1155/2013/572301

**Published:** 2013-04-03

**Authors:** Peter Chau, Jeremy Moore

**Affiliations:** ^1^Department of Pediatrics, UCLA Mattel Children's Hospital, Los Angeles, CA 90095, USA; ^2^Department of Pediatrics, Division of Pediatric Cardiology, UCLA Mattel Children's Hospital, 10833 Le Conte Avenue, B2-427 MDCC, Los Angeles, CA 90095, USA

## Abstract

The case of a 14-year-old female with ectopic atrial tachycardia who had been followed for a history of anxiety and depression is presented. The patient was admitted to the intensive care unit after she attempted suicide, at which point there was electrocardiographic evidence of the atrial arrhythmia. During subsequent invasive electrophysiology study, a focus near the tricuspid annulus was localized and targeted for ablation, followed by resolution of the psychiatric symptoms. The case highlights the overlap between psychiatric complaints and true cardiac arrhythmia. A review of the literature is presented, with a special emphasis on distinguishing these two entities as well as a synopsis of this uncommon arrhythmia in the pediatric population.

## 1. Case Presentation

The patient is a 14-year-old female with no other significant medical history until she began experiencing several life stressors that gradually escalated prior to her suicide attempt with multiple tablets of Lorazepam and alcohol. Ten months prior to admission after learning of a maternal medical illness, she began feeling depressed and anxious and experienced “panic attacks” which included palpitations, dizziness, and occasional blurred vision. She was unable to sleep and experienced decreased appetite, weight loss, anhedonia, social withdrawal, impaired concentration, and emotional numbing. Her “panic attacks” and palpitations were characterized as intense and brief, lasting for several seconds. Her symptoms occurred once a week, but eventually progressed to multiple times a week. She later sought psychiatric evaluation after experiencing suicidal ideations and began cutting her wrists. This prompted psychotherapy, and she was prescribed Ativan and Zolpidem to assist with sleep.

**Figure 1:**
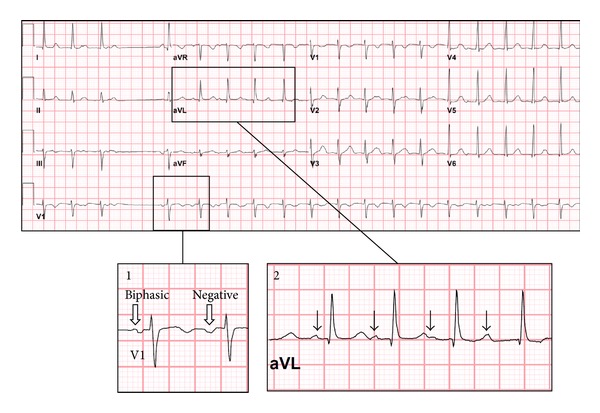
Initial ECG obtained in the pediatric ICU. Ectopic atrial tachycardia is present that is characterized by spontaneous termination and reinitiation, with a single low atrial escape beat before reinitiation (inset 1). Notice the change in P wave axis in lead V1 from biphasic to completely negative upon transition from the escape beat to the ectopic rhythm (white arrows), a finding highly suggestive of right atrial tachycardia [1]. This is followed by progressive prolongation of the PR interval until the P wave is obscured by the T wave (inset 2, black arrows), which is also common with this arrhythmia mechanism. The rhythm was slowest at the time of diagnosis, likely due to the large dose of benzodiazepine that had been ingested prior to admission.

**Figure 2:**
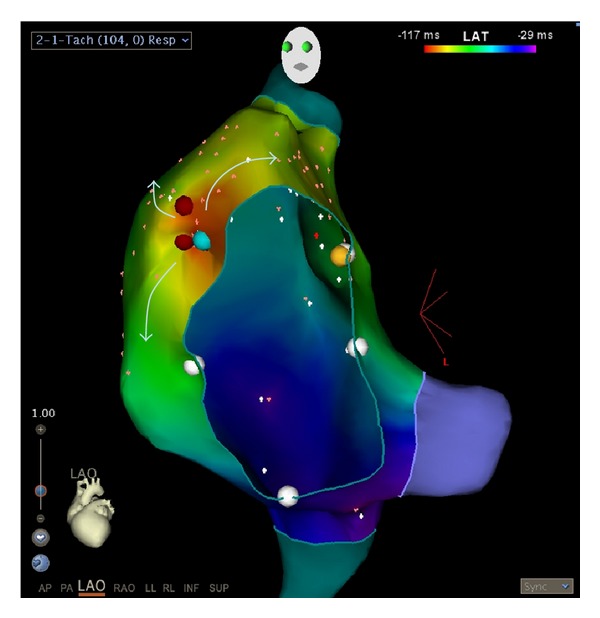
Three-dimensional activation map of the right atrial tachycardia. The tricuspid annulus is viewed “en face” from the LAO view. The earliest activation is depicted in red with concentric spread depicted by colors of yellow, green, blue, and magenta (arrows). An early focus was encountered at the 10 o'clock position of the tricuspid annulus (light blue marker), where a radiofrequency lesion was placed, terminating the tachycardia (red marker). A consolidation lesion was also placed at this site (red marker).

After her suicide attempt, she was admitted to the intensive care unit. She was persistently tachycardic for which an ECG was obtained (Figure 1). This demonstrated an ectopic atrial tachycardia (EAT) suspected to be due to a focus in the high lateral right atrium. Her echocardiogram revealed mild left ventricular dilation with a depressed ejection fraction of 40%. She was transferred to the inpatient psychiatric ward service for 4 weeks with ongoing cardiology consultation. Fluoxetine was initiated, but she continued to have feelings of anxiety, depression, and palpitations for the next few weeks although her symptoms improved with both psycho- and pharmacologic therapy. After medical and psychiatric stabilization, she underwent catheter ablation which confirmed the diagnosis of EAT (Figure 2). Immediately after the ablation, her feelings of anxiety and depression dramatically improved and she has since had no further episodes of depression or suicidal ideation. She is now doing well in school and her relationship with her family has stabilized. She has continued taking Fluoxetine without side effects and continues to have regular visits with her psychologist. Followup echocardiography revealed normalization of ventricular function.

## 2. Discussion

### 2.1. Distinction between Psychiatric Disturbance and Arrhythmia

The symptoms associated with psychiatric illnesses and arrhythmia oftentimes overlap, frequently creating problems for both diagnosis and therapy. These symptoms (among others) include palpitations, dizziness, chest pain, diaphoresis, and anxiety. Not only may psychiatric illness present with symptoms suggestive of arrhythmia, but nearly as frequently, arrhythmia can present with symptoms suggestive of psychiatric disturbance. Approximately 90% of patients with panic disorder experience palpitations and of those, 25% are initially referred for cardiac evaluation before treatment of the psychiatric disorder [2, 3]. It is also estimated that more than half of patients diagnosed with paroxysmal supraventricular tachycardia (PSVT) previously had symptoms ascribed to panic disorder, stress, or anxiety and greater than two-thirds with clinically confirmed PSVT meet DSM-IV criteria for panic disorder, adding to the confusion [4]. Finally, the combination of true psychiatric and cardiac pathology can occur simultaneously in up to 4% of cases which may explain why patients with PSVT may continue to experience palpitations and panic disorder symptoms after catheter ablation [4]. Usually however, psychologic functioning and quality of life improve following catheter ablation for supraventricular arrhythmia [5].

Although physical exam findings in the vast majority of cases are unremarkable, these should be carefully sought by the experienced physician. Incessant tachycardias may result in physical exam findings of congestive heart failure. Jugular venous distention with cannon waves can be noted in patients with congenital junctional ectopic tachycardia or ventricular arrhythmia. Grouped beating (consecutive conducted ventricular complexes followed by brief interruption before resumption) suggests an atrial tachycardia with Wenckebach conduction, but can also be seen with the persistent form of junctional reciprocating tachycardia in which spontaneous termination and reinitiation are common. A screening ECG is useful if abnormal but is not a sensitive indicator of intermittent arrhythmia. In most cases, rhythm documentation during the patient's symptoms is vital to the diagnosis and if intermittent, ambulatory monitoring with an event monitor is recommended. Monitoring may either diagnose PSVT during the times of anxiety or palpitations or lend support to its absence when symptoms consistently occur during documented sinus rhythm.

The onset of the episode should be carefully explored, as a sudden increase in heart rate, at times initiated by a skipped beat, is highly suggestive of PSVT. The quality of the palpitations should also be characterized, as the sensation of forceful but not particularly rapid heart beat is more suggestive of anxiety than a rapid and sustained rhythm that is “too fast to count.” Oftentimes asking the older child to tap out the heart beat with their hand can clarify this issue. “Shirt flapping” or regular rapid neck pulsations may be associated with PSVT, especially those types that are associated with contraction of the atria against a closed AV valve resulting in reflux of blood into the SVC [6]. Among the various physical exam findings, it has been shown that regular rapid pounding neck sensation or visible neck pulsation is one of the most sensitive and specific for PSVT and should not be ignored [7]. 

### 2.2. Ectopic Atrial Tachycardia

EAT is usually idiopathic and seen in patients with structurally normal hearts. It comprises a minority of patients with supraventricular arrhythmia and is observed less commonly in children. In EAT, the nonsinus atrial focus usually generates a narrow complex tachycardia with visible P waves at an inappropriately rapid rate. The P axis commonly is distinct from that of sinus rhythm unless the ectopic focus is very near the sinus node. The average heart rate ranges from 110 to over 200 bpm but varies during the course of a day and can rarely exceed 300 bpm under intense sympathetic stimuli [8]. The variability of heart rate is important in differentiating between ectopic atria tachycardia and other reentrant forms of SVT [8].

Long-term efficacy of pharmacologic therapy may be poor, and many advocate radiofrequency ablation as first-line therapy. Because a high percentage of patients with EAT progress to cardiomyopathy, catheter ablation is particularly appealing [9]. Fortunately, the cardiomyopathy often reverses with resolution of the arrhythmia, and this is especially true in children [10, 11]. It is highly effective, safe, and associated with low recurrence rates [12]. In the present case, catheter ablation played a vital role in the patient's overall emotional improvement as well as normalization of her ventricular function, both of which would have likely further deteriorated over time.

### 2.3. Psychotropic Medications and Screening ECG

The use of psychotropic medications has increased in recent years. Cases of sudden death have been noted particularly with tricyclic antideprssants (TCAs), which has raised concern since several psychotropic medications have a propensity to prolong the QT interval and result in conduction abnormalities. To date, there is no direct evidence linking prior deaths to the effects of the drugs on the QT interval [13]. Nevertheless, the American Heart Association has recommended that all children be screened with an ECG before starting a TCA or the antipsychotic medications of haloperidol, pimozide or phenothiazine [14]. For other psychotropic medications, a screening ECG is recommended if a patient is at an increased risk of arrhythmia. The clinician should focus on symptoms such as palpitations and syncope as well as any family history of congenital heart disease, QT prolongation, sudden death, unexplained syncope, or seizures. Any medications that may interact with psychotropic medications should also be noted. This assessment is to make certain that there is no predisposition to arrhythmia before starting psychotropic medication in the pediatric population.

## 3. Summary

There is limited data linking psychiatric disorders with arrhythmia. This case underscores the need to screen patients for arrhythmia when being evaluated by their general pediatrician or psychiatrist for psychiatric illness. This patient had an underlying EAT concomitant with her underlying anxiety and depression. Prompt diagnosis may have prevented the long-term sequelae of recurrent psychiatric illness and/or tachycardia-induced cardiomyopathy. Immediately after ablation, her anxiety and depression improved and she has since been asymptomatic without recurrence—now over a year since her procedure. She has continued Fluoxetine and has been followed closely by her psychiatrist.
